# Iwilfin (eflornithine) approved by the FDA as the first and only oral maintenance therapy for high-risk neuroblastoma in adult and pediatric patients: Narrative review

**DOI:** 10.1097/MD.0000000000040662

**Published:** 2024-11-29

**Authors:** Ayesha Shakeel, Aniqa Baloch, Versha Kumari, Syeda Kainat Zehra Kazmi, Kanza Aftab, Shiza Abid, Amna Syed, Juvairia Yousuf, Muhammad Hasanain, Muhammad Umair Anjum, Mohammed Mahmmoud Fadelallah Eljack

**Affiliations:** a Department of Internal Medicine, Jinnah Sindh Medical University, Karachi, Pakistan; b Department of Medicine and Surgery, Dow University of Health Sciences, Karachi, Pakistan; c Department of Medicine and Surgery, Ayub Medical College, Abbottabad, Pakistan; d Faculty of Medicine and Health Sciences, University of Bakht Alruda, Ad Duwaym, Sudan.

**Keywords:** DFMO, Iwilfin, neuroblastoma, neuro-oncology, pediatric

## Abstract

Neural crest progenitor cells give rise to neuroblasts, the growing nerve cells of the sympathetic nervous system. These cells can undergo changes leading to neuroblastoma, a malignancy responsible for 15% of all pediatric cancer-related deaths. The molecular pathogenesis of this pediatric cancer involves complex genetic alterations, such as MYCN amplification, chromosomal abnormalities, and gene expression changes. Despite aggressive therapies, survival rates for children with high-risk neuroblastoma (HRNB) have not improved significantly compared to those with less severe forms of the disease. This highlights the challenge of managing HRNB and underscores the need for new, effective treatments. A comprehensive treatment regimen, including immunotherapy, radiation therapy, myeloablative chemotherapy, and surgical removal, has been employed to achieve remission in HRNB patients. While dinutuximab beta immunotherapy is an effective and widely used treatment, it has several potential side effects that must be carefully monitored. New drugs are being developed to reduce these side effects without compromising efficacy. One such drug is DL-alpha-difluoromethylornithine (DFMO), approved by the FDA under the brand name Iwilfin. Numerous clinical trials have shown that DFMO, when used as maintenance therapy, significantly improves event-free survival and overall survival in neuroblastoma patients. However, DFMO has adverse effects that require continuous monitoring. Further research is needed to minimize these side effects and improve its efficacy, particularly in addressing resistance caused by long-term use.

## 1. Introduction

Neuroblastoma is a malignancy of the autonomic nervous system that develops from progenitor cells generated from neural crest tissues.^[1]^ Usually located in the paraspinal ganglia or adrenal medulla, these tumors present as masses in different areas like the chest, neck, pelvis, or abdomen. Clinical presentations vary widely, ranging from asymptomatic masses to locally invasive or widely disseminated tumors. While some patients may show no indications, others may experience symptoms such as a bulging mass, discomfort, and compression/hepatomegaly causing abdominal distention or respiratory difficulty. Other possible constitutional symptoms include fever, malaise, lymphadenopathy, and weight loss. Thoracic involvement may lead to difficulty eating, breathing, or, rarely, thoracic outlet syndrome, while pelvic tumors can cause dysuria and constipation. Cervical tumors may give rise to classic triad of miosis, ptosis, facial anhidrosis, and epidural extension may result in neurological deficits such as a progressive loss of motor function in approximately 15% of instances. Other congenital disorders such Hirschsprung disease, hypoventilation disorder, and neurofibromatosis type 1 can coexist with neuroblastoma.^[2]^

Neuroblastoma is responsible for 15% of all cancer-related deaths in pediatric population.^[2]^ Approximately 10.2 cases per million children under 15 years old are diagnosed with high-risk neuroblastoma annually, totaling nearly 500 new cases each year.^[3]^ With a mean diagnostic age of 22 months, 90% of diagnosis happens before the age of 5, and of those 30% of diagnosis happen during the first year.^[3]^ The outcomes in adolescence and adulthood are noticeably worse, even though incidents in these age ranges are uncommon. There seems to be a little male predominance (1.2:1), but no apparent racial propensity.^[4]^

Family history is observed in 1% to 2% of the cases, and autosomal dominant inheritance patterns have been reported.^[3]^ In the last thirty years, there has been a constant improvement in the prognosis for neuroblastoma patients, as evidenced by a rise in 5-year survival rates from 52% to 74%.^[5]^ However, this improvement primarily reflects increased cure rates among those with the less severe form of the disease. Survival rates for children with high-risk neuroblastoma have seen only marginal improvement despite more aggressive treatment strategies.^[5]^

Neuroblastoma survivors have significant concerns with regard to long-term morbidity and death. Late treatment-related side effects often include stunted growth, kidney problems, underactive thyroid, hearing loss, cognitive decline, and the development of secondary cancers like acute myeloid leukemia.^[6]^ Radiation therapy to the head poses an increased risk of blindness, particularly when the disease involves the base of the skull or the orbits and is treated with radiation therapy.^[6]^ While neuroblastoma survivors may achieve similar levels of educational attainment as their siblings, they often experience disparities in psychosocial outcomes, such as lower employment rates, reduced individual and household income, and decreased likelihood of marriage, indicative of reduced social integration.^[7]^

### 1.1. Pathogenesis

Neuroblasts are developing nerve cells derived from neural crest progenitor cells. Neuroblastoma occurs as a result of transformation of these immature sympathetic nervous system cells. This pediatric cancer has a complex molecular pathophysiology that includes genetic changes such as MYCN amplification, segmental chromosomal changes, and gene expression profiling.^[8]^ Newer research has also revealed important genetic markers linked to low-risk forms of neuroblastoma, including anaplastic lymphoma kinase gene (ALK) mutations and genomic variants in genes including DUSP12, DDX4, IL21RA, and HSD17B12.^[9]^ The oncogenesis of neuroblastoma has also been linked to changes in microRNA expression and epigenetic perturbations.^[8]^

In conclusion, a complex interplay of genetic changes, such as MYCN amplification, segmental chromosomal modifications, gene expression profiles, ALK mutations, and other genomic variants, contributes to the complex pathogenesis of neuroblastoma.

### 1.2. Classification

Peripheral neuroblastic tumors include neuroblastoma and its subtypes, intermixed ganglioneuroblastoma, ganglioneuroma, and nodular ganglioneuroblastoma.^[10]^ The degree of neuroblastic differentiation (undifferentiated, weakly differentiated, and differentiating) and the mitosis-karyorrhexis index (low, middle, or high) can be used to further categorize neuroblastoma.^[10]^

The International Neuroblastoma Staging System when developed, took into account factors such as the extent of tumor excision, the involvement of either the ipsilateral or contralateral lymph nodes, tumor spread across the body’s midline, and the discrimination between stage 4S and stage 4. However since it is determined only after surgery, it is not a useful tool to be used in children especially in cases where they did not need or cannot have surgery.^[11]^ In contrast, the International Neuroblastoma Risk Group Staging System (INRGSS) is determined before surgery through imaging investigations, examinations, and biopsies rather than incorporating surgical outcomes as a part of its grading process. INRGSS is now used for almost all neuroblastoma tumors as it is based on risk factors defined by imaging and clinical work-up.^[12,13]^

### 1.3. High-risk neuroblastoma

Prior to treatment patients are classified using the INRG according to various risk factors. These variables encompass INRG imaging stage, age, and pathology. Poorer outcomes are associated with the occurrence of segmental chromosomal abnormalities, age >18 months, and disease that has progressed to advanced stage.^[14]^ The MYCN oncogene, identified in around 25% of neuroblastoma cases, is strongly associated with a poor prognosis and consistently indicates high-risk status regardless of circumstances.^[3]^ Mutations in the ALK gene, occurring in roughly 5% to 10% of neuroblastoma cases, are linked to diminished overall survival (OS).^[4]^ Treatment modalities for high-risk neuroblastoma (HRNB) are continuously evolving and typically entail immunotherapy, autologous stem cell transplantation (ASCT), intensive chemotherapy, and radiation therapy. Presently, research endeavors are concentrated on devising innovative strategies to enhance outcomes for these challenging patients, minimizing toxicity and late side effects, and identifying more personalized treatment approaches. The objective of this study is to delineate the advantages and importance of the recently approved medication for HRNB in comparison to established treatment modalities.

## 2. Methodology

A comprehensive literature search was conducted on Google Scholar and PubMed using the keywords “Iwilfin” and “Neuroblastoma.” We also searched for “Eflornithine.” Studies carried out on children with recurrent or high-grade neuroblastoma were included. The studies and their findings were discussed in detail. For Eflornithine, its mechanism of action and usage was noted down. For Neuroblastoma, a comprehensive literature search was carried out to note down the current treatment regimen approved for it. Table 1 below mentions some of the commonly used abbreviations.

**Table 1:** Key abbreviations used in this review.

Abbreviation	Description
DFMO	Difluoromethylornithine
BCC	Beat Childhood Cancer Consortium
EFS	Event-free survival
OS	Overall survival
HRNB	High-risk neuroblastoma

No ethical approval or consent for participation was required for this review.

## 3. Treatment

For patients with HRNB, a comprehensive treatment plan that includes therapies including immunotherapy, therapy with radiation, myeloablative chemotherapy, and surgical removal procedures has been used to induce remission. Due to the unique combination and intensity of treatments required for each patient, follow-up protocols have to be tailored accordingly. The majority of hospitals in North America and worldwide collaborative groups for pediatric oncology adhere to a treatment approach that includes chemotherapeutic induction and surgical resection of the main tumor. Following radiation treatment and consolidation with ASCT, post-consolidation therapy consists of isotretinoin and anti-ganglioside 2 (GD2) immunotherapy. The duration of the upfront therapy is around 18 months.^[15]^

### 3.1. Induction

Eradication of visible illness and attainment of remission are the objectives of induction. Intensive chemotherapy comprising alkylators, platinum, and topoisomerase medications is usually administered in 5 to 8 cycles as part of induction protocols.^[15]^ A high-dose induction chemotherapy regimen was utilized for 24 newly diagnosed high-risk neuroblastoma (NB) pediatric patients participating in the N6 protocol at the Memorial Sloan Kettering Cancer Center (MSKCC), incorporating a multi-modal approach. Research conducted in 1994 detailed the antitumor and toxic consequences of this regimen.^[16]^ Based on the Goldie–Coldman theory, the induction’s objective was to combine 2 pharmacological combinations that are thought to be non-cross-resistant: high-dose cisplatin/etoposide (P/E) and high-dose cyclophosphamide + doxorubicin/vincristine (CAV).^[17]^ Later MSKCC multimodality regimens (N7 and N8) used chemotherapy with the same induction. It was discovered that this chemotherapy treatment maintained a good response rate and had less toxicity with 2 modifications: 5 cycles (instead of 7) and a lower vincristine dosage.^[18]^ This was predicated on an additional 63 patients’ follow-up analysis (for a total of 87).^[18]^ In comparison to MSKCC cycles, mucositis incidence was reduced following topotecan cycles and 84% of patients had a tumor response, according to a study intended to examine the feasibility of including cyclophosphamide and dose-intensive topotecan into induction therapy.^[19]^ Five rounds of topotecan, vincristine, doxorubicin, cyclophosphamide, cisplatin, and etoposide are used in the current COG studies.^[15]^ A different study contrasted the quick COJEC regimen (rapid cisplatin, vincristine, carboplatin, etoposide, and cyclophosphamide) with the Memorial Sloan Kettering N5 regimen. Although rCOJEC and MSKCC-N5 did not significantly vary in their outcomes, rCOJEC showed reduced acute toxicity. Thus, for the European Neuroblastoma Group of the International Society of Paediatric Oncology, rCOJEC is the recommended induction regimen.^[20]^

After many rounds of chemotherapy, the primary tumor is often surgically removed during induction. An essential part of upfront therapy is surgery. This method minimizes surgical morbidity by enabling optimum tumor shrinking.^[15]^ A substantial increase in survival was observed in patients diagnosed with advanced high-risk neuroblastoma in the HR-NBL1/SIOPEN trial who underwent surgeon-assessed CME of the original tumor.^[21]^ Achieving a surgeon-assessed resection extent of more than 90% seems to result in a favorable treatment outcome in terms of reducing the incidence of local recurrence overall and maybe enhancing event-free survival (EFS).^[21]^ By the time induction ends, 10% of patients will have deteriorating disease despite the rigors of the induction protocol, and just 20% will show full recovery. Since the end-of-induction response is predictive of the outcome, as several studies have demonstrated, the updated 2017 International Neuroblastoma Response Criteria have been validated.^[22]^

### 3.2. Consolidation

High-risk neuroblastoma patients receive consolidation therapy following the induction phase of treatment to eradicate any residual disease. During intensification phase of high-risk neuroblastoma treatment, patients typically undergo 2 rounds of intense chemotherapy, followed by a procedure where their own stem cells are harvested and then transplanted back into their body. During ASCT, the individual undergoes treatment with high dose of chemotherapy leading to considerable myelosuppression. Following this, their preserved stem cells are returned to them to stimulate bone marrow recovery.^[15]^ North America and Europe use different regimens for ASCT in patients with HRNBL. The Americans typically use carboplatin, etoposide, melphalan while the Europeans utilized Bu/Mel (busulfan/melphalan).^[23]^ Tandem cell transplants (2 back to back stem cell transplants) is better than single stem cell transplant as suggested by new studies.^[24]^ After ASCT, a dose of 21.6 Gy of external beam radiation therapy is administered to target any remaining primary tumor and persistent metastatic lesions at the conclusion of the initial treatment phase.^[25]^ To reduce radiation exposure to adjacent organs without increasing recurrence rates, proton radiation therapy was better than photon therapy as suggested by new studies.^[26]^ Although radiation is essential for local management in neuroblastoma, which is a radiosensitive disease, research on radiation reduction has been spurred by concerns about the enduring negative impacts of radiation. Overall, the intensification phase of high-risk neuroblastoma treatment is a critical component of the multi-modal approach that has led to improved survival rates for these patients in recent years.

### 3.3. Post-consolidation

The aim of the maintenance phase is to eradicate any remaining disease and prevent relapse of aggressive neuroblastoma. In addition to other supportive therapies like isotretinoin and granulocyte-macrophage colony-stimulating factor (GM-CSF), it might involve monoclonal antibody therapy, such as dinutuximab. Dinutuximab, a monoclonal antibody against GD2, which is highly expressed on the outer surface of neuroblastoma cells is 1 targetable approach. It is administered in conjunction with cytokines such as GM-CSF to enhance the immune response against malignant cells.^[27]^ Since GD2 is also found on peripheral nerves as well, treatment with GD2 antibodies is associated with high levels of neuropathic pain. Fever, capillary leak and allergic reaction are other side effects.^[15]^ It has been demonstrated that administering isotretinoin, sometimes referred to as 13-cis-retinoic acid, during the maintenance stage of high-risk neuroblastoma treatment improves outcomes. It is thought to aid in differentiation of any residual neuroblastoma cells, hence making them less aggressive.^[28]^ The cytokine GM-CSF is also administered to boost the immune system response by boosting T cells that attack cancer, particularly monocytes and macrophages.^[27]^ In general, the combination of monoclonal antibody therapy, cytokine support, and isotretinoin has become a standard part of the maintenance treatment for high-risk neuroblastoma patients.

Treatments currently being studied include targeted therapies such as loratinib, crizotinib, and adavosertib to treat relapsed neuroblastoma that has mutations in the ALK gene and radiotherapy compromising of iodine 131-MIBG therapy, which uses radioactive iodine to target neuroblastoma cells, which is being studied alone or in combination with chemotherapy for refractory disease.^[27]^

### 3.4. Immunotherapy adverse events

However, certain adverse consequences have been reported to be associated with administering this immunotherapy. The majority of adverse effects are temporary and disappear during the course of therapy. Some of the most frequent side effects reported include neuropathic pain (52%), infection (39%), fever (39%), capillary leak syndrome (23%), hypersensitivity reaction (25%), hyponatremia (23%), hypokalemia (35%), transaminitis (severely elevated alanine aminotransferases and aspartate aminotransferases levels; 23%), gastrointestinal side effects (vomiting and diarrhea; 22%), hypotension (18%), hypoxia (13%), and urticarial.^[29]^ Fever, hypotension, and capillary leak syndrome are a few examples of adverse events that can be challenging to diagnose and differentiate from sepsis and infections. The underlying reason may also have a significant impact on how these symptoms are managed. When symptoms do not go away quickly after discontinuing dinutuximab beta or following appropriate supportive care, other adverse events that might be connected to NB itself should be taken into consideration. Children experiencing a relapse in their condition may exhibit symptoms related to the central nervous system or bone discomfort.^[30]^

Except for life-threatening circumstances, corticosteroids have to be avoided during the dinutuximab beta treatment period, which runs from 2 weeks before the first dosage in the first cycle to 1 week following the last dose in the last cycle. Corticosteroids are known to interfere with immunotherapy because of their immunosuppressive properties, yet there has not been any research done to confirm this.^[31]^ Dinutuximab selectively binds to the disialoganglioside GD2, which is expressed on the surface of neuroblastoma cells as well as peripheral sensory nerve fibers and neurons. Consequently, neuropathic discomfort is experienced by patients during dinutuximab infusions. Although patients can feel discomfort anywhere in the body, the abdomen is the most common location.^[29]^ Fever is also a common adverse effect of dinutuximab-based therapy that is caused by the body’s immunological response. Fevers can happen at any point throughout the dinutuximab infusion, even hours after it has finished. Acetaminophen is frequently administered in advance of administration of dinutuximab since fever is a common side effect of antibody therapy. Patients frequently continue to have high fevers after this intervention, which might occasionally worry nurses as well as the patient and his or her family. If acetaminophen is not able to bring down the fever, ibuprofen may be given if the patient’s platelet count allows it and institutional practice dictates it. Ibuprofen is frequently beneficial.^[32]^ Dinutuximab, IL-2, or a combination of the 2 may have toxic effects that are responsible for hypersensitivity reactions. Although hypersensitivity responses have been observed to occur more frequently during IL-2-containing courses, they can occur throughout any cycle including dinutuximab.^[2]^ It has been shown that capillary leaks happen more frequently in courses that contain IL-2 than in courses that contain GM-CSF.^[29]^ Possible symptoms include decreased urine output, hypotension, decreased intravascular volume, and widespread edema, either alone or in conjunction with pulmonary edema and ascites. Patients are scrupulously recorded for intake and output, their weight is taken twice a day, and IV fluid rates are titrated based on condition because fluid shifts may occur. Hyponatremia from capillary leak syndrome is possible, but it is usually not severe enough to require an injection of hypertonic saline. Every day, or more frequently, if necessary, electrolytes should be checked.

Patients who use drugs for uncontrollably high pain may encounter hypoxia if they develop hypersensitive reactions, capillary leaks, or oversedation. When using dinutuximab, continuous pulse oximetry monitoring is necessary.^[32]^ Despite being less emetogenic than chemotherapy, immunotherapy can nevertheless cause nausea and vomiting in certain patients for unknown reasons.^[32]^ For patients with high-risk NB, SIOPEN has recommended dinutuximab beta immunotherapy as part of the standard of care.^[33]^ However, given the adverse safety profile, newer interventions have been in the process which would reproduce the same or a better result with lesser chances of undesirable consequences.

### 3.5. Efornithine

Approximately 4 to 5 decades ago, DL-alpha-difluoromethylornithine, commonly known as DL-alpha-difluoromethylornithine (DFMO), was found for its ability to block ornithine decarboxylase (ODC), an enzyme required for the manufacture of polyamines like spermine, spermidine, and putrescine. Polyamines play a pivotal function in cell growth and apoptosis suppression via multiple cellular signaling pathways.^[34]^ Additionally, DFMO targets the oncogene MYCN, which can undergo mutations causing normal cells to undergo a malignant change. By blocking these pathways, DFMO suppresses the development and growth of neuroblastoma tumors.^[35]^

Initially developed as an anticancer drug, DFMO garnered attention for its effectiveness in treating African trypanosomes, leading to its approval by the FDA in 1990 for the management of African sleeping sickness.^[4–10]^ For almost 50 years, eflornithine, the active component of DFMO, has been approved therapy for this illness.^[36]^ The efficacy of DFMO in targeting ODC was further demonstrated in studies showing its ability to suppress putrescine production in parasites and eradicate parasites in animal models.^[37]^

However, despite its promising therapeutic effects, DFMO’s high dosage requirements and substantial production costs led to a decline in its use in Africa during the 1990s.^[38]^ Nevertheless, the FDA approved a DFMO cream, marketed as Vaniqa, in 2000 for the treatment of hirsutism.^[39]^ Although DFMO has shown promising results in clinical trials for colon cancer, it has yet to receive FDA approval for this indication.^[40–44]^

Recent research has highlighted the correlation between ODC expression and MYCN gene expression in neuroblastomas, demonstrating DFMO’s promise as a targeted treatment for this cancer.^[40]^ A research conducted in 2008 by Hogarty et al found that ODC1 expression is substantially greater in neuroblastomas that are positive for MYCN and that there is a very significant connection (*R* = 0.80, *P* < .0001) between MYCN expression and ODC1 expression. Higher ODC1 expression in tumors was linked to worse EFS and OS.^[41]^ Clinical studies have demonstrated that DFMO, when administered as maintenance therapy, effectively increases EFS by nearly 30%, from an average of 55% to approximately 85% in pediatric neuroblastoma patients, emphasizing the importance of targeting ODC in cancers with dysregulated polyamine metabolism and Myc mutations.^[40]^

On 13th December 2023, the FDA approved eflornithine, under the brand name IWILFIN, with the goal of lowering the likelihood of recurrence in patients with HRNB, both adult and pediatric, who responded partially to previous multiagent, multimodality treatment including immunotherapy against GD2.^[42]^

## 4. Trial results

Numerous clinical trials have been carried out to assess the safety and efficacy profile of this medication on the patient population. From June 2012 through to February 2016, patients with HRNB participated in a phase 2, open-label, single-agent, multicenter clinical trial. It was authorized by institutional review boards in 22 cities and enrolled patients with histologically confirmed neuroblastoma classified as high-risk under the International Neuroblastoma Risk Group Classification. Patients in stratum 1 had completed 5 to 6 cycles of chemotherapy according to “COG,” “MSKCC” or “SIOPEN” regimens while children in stratum 2 were eligible for participation if they had finished any relapsed or augmented therapy for primary refractory illness. The patients were prescribed Iwilfin 250 mg tablets (oral DFMO) for 27, 4-week cycles. The dosage was adjusted to 750 ± 250 mg/m^2^ dose, twice daily. The primary endpoint was EFS from the first dose of DFMO while secondary endpoints included OS and safety assessments. Monthly physical examinations, adverse event assessments, and laboratory evaluations were conducted throughout the trial period. The endpoints were evaluated using Kaplan–Meier curves. Among all subjects who received DFMO in stratum 1, two-year EFS was 84% with an OS rate of 97%. In stratum 2, two-year EFS was 51% with an OS rate of 84%. Patients with original refractory illness had an EFS of 68% and OS of 89% while previously relapsed patients had an EFS of 35% and OS of 80%. Hence, the administration of DFMO to children with HRNB following completion or relapse of therapy demonstrated improved rates of EFS and OS.^[43]^

A subset analysis was conducted on 81 of the 100 individuals enrolled in the preceding trial. These were the patients who had received the standard therapy as per the COG guidelines followed by maintenance DFMO. Their outcomes were compared to a retrospective cohort of individuals receiving the same treatment but without DFMO in Beat Childhood Cancer Research Consortium hospitals. The control group comprised 76 patients. The results of this subset analysis revealed statistically significant differences in both the EFS and OS. The 2 and 5-year probabilities for EFS in the group treated with DFMO were 86.4% and 85.2%, respectively. In contrast, the control group exhibited probabilities of 78.3% and 65.3% at 2 and 5-years respectively. The 2 and 5-year OS for the DFMO group were 98.8% and 95.1% while for the control group, the probabilities were 94.4% and 81.6%. Therefore, the analysis showed that children who underwent DFMO following conventional therapy demonstrated a substantial improvement in EFS and OS.^[44]^

Another retrospective analysis was carried out in patients with high-grade neuroblastoma. Patients enrolled in the NMTRCOO3B trial who received DFMO were compared to patients enrolled in the ANBL0032 trial, who did not receive DFMO post-immunotherapy. The primary endpoint for this study was EFS while the secondary endpoint was OS. The patients were analyzed using an unadjusted Cox proportional hazards model, using treatment (DFMO vs non-DFMO) as the only control. Furthermore, based on 11 baseline covariates: including age at high-risk diagnosis, sex, race, stage at HRNB diagnosis according to the 1993 International Neuroblastoma Staging System: pre-ASCT response, transplant type, time from ASCT to initiation of immunotherapy, duration of immunotherapy, overall response at the end of immunotherapy, time from diagnosis to the end of immunotherapy, and MYCN amplification status-propensity score matching was used to equilibrate DFMO and non-DFMO patients. The matched cohorts were then compared using unadjusted Cox proportional hazards models controlling only for treatment. In the overall population, significant enhancement in EFS was observed in the DFMO group, with a rate of 84% compared to 72% in the non-DFMO group. Four-year OS rates for the DFMO group were 96%, contrasting with 84% in the non-DFMO group. Similar trends were observed in the matched cohorts analyzed through propensity score matching. The four-year EFS was 84% versus 73% in the non-DFMO group. The four-year OS rates also showed a similar trend favoring DFMO use with rates of 96% in the DFMO group, compared to 84% in the non-DFMO group. All findings were statistically significant.^[45]^

Lastly, we decided to compare the outcomes of the clinical trials conducted for Iwilfin with those of the conventional treatment regimen administered for high-grade neuroblastoma. Patients receiving typical postoperative immunotherapy, which included isotretinoin, GM-CSF, IL-2, and dinutuximab, had a 2-year equivalent survival rate of 66%,^[45]^ whereas those receiving DFMO demonstrated a notably higher rate (86.4%).^[33]^ Similarly, the 2-year OS rate for the former group stood at 84%^[29]^ whereas for the latter group, it soared to 98.8%.^[44]^ Consequently, the utilization of DFMO exhibited enhanced survival outcomes in contrast to the conventional treatment regimen.

Currently, Iwilfin is being considered for use in post-immunotherapy or as a maintenance agent with the standard treatment of care for high-grade neuroblastoma. Among the most common side effects of using Iwilfin include upper respiratory tract infections, sinusitis, otitis media, cough, sinusitis, pneumonia, and diarrhea. Reduced neutrophils, hearing loss, higher alanine aminotransferase and aspartate aminotransferase, as well as an increased risk of skin and urinary tract infections, are other possible outcomes.^[42]^ Due to this, patients may find it difficult to follow the recommended treatment plan accounting to how frequently and how severe these side effects occur. Regular monitoring is essential to assess and manage any side effects that may occur from Iwilfin, which poses practical challenges for both patients and healthcare providers. Therefore, it is wise to suggest careful monitoring and thorough counseling in order to properly address and manage these possible adverse effects. In addition, people can face difficulties in affording or obtaining Iwilfin therapy, especially in areas with underdeveloped healthcare systems. Iwilfin is not advised for people with renal impairment and bears the risk of interfering with many other drugs.^[46]^ Patients may become resistant to Iwilfin over time, which would decrease the medication’s efficacy. Table 2 provides a summary of the outcomes reported in the clinical trials referenced in this study.

**Table 2:** Summary of trials.

S. no.	Clinical trial	Type of clinical trial	Total number of participants	Trial assessment	Trial duration	Results
1	SHOLLER	Multicenter, open-label, single-agent, phase 2	100	Event-free survival, overall survival and safety	3 years and 8 months.	Stratum 1: EFS: 84%, OS: 97%. Stratum 2: EFS: 51%, OS: 84%
2	LEWIS	Subset analysis of the Sholler clinical trial.	81 + 76 in the control group	Event- free survival and overall survival	3 years and 8 months.	After 2 years: EFS: 86.4, OS: 98.8. After 5 years: EFS: 85.2, OS: 95.1%
3	OESTERHELD	Retrospective analysis of a clinical trial	852 + 87 + 5	Event-free survival and overall survival	Follow-up for DFMO patients: 6.1 years, Non-DFMO: 5 years.	4-year EFS: 84%, OS: 96%

DFMO = difluoromethylornithine, EFS = event-free survival, OS = overall survival.

## 5. Discussion and future prospects

Due to its wide range of clinical symptoms, intricate molecular pathogenesis, and unpredictable response to therapy, neuroblastoma poses a great challenge in pediatric oncology. This paper highlights that although there have been notable developments in treatment strategies in recent years, high-risk neuroblastoma still presents considerable clinical and scientific difficulties. Neuroblastoma accounts for a high number of cancer-related deaths in children, thus establishing effective therapies is critical. MYCN amplification, segmental chromosomal alterations, gene expression patterns, ALK mutations, and other genomic abnormalities all contribute to neuroblastoma’s complicated pathology. These factors help predict disease progression and prognosis. International Neuroblastoma Staging System and INRGSS are classification systems that assist clinicians in making treatment decisions while also predicting illness prognosis.

Nonetheless, since different studies have different views about the prognostic importance of particular genetic markers or classification systems and because new genetic markers and treatment approaches are being discovered, validation studies should be carried out to keep treatment approaches and categorization systems up to date. Multiple clinical trials have been conducted to assess the safety and effectiveness of DFMO. Studies have also found that treated patients have improved EFS and OS. Because of these favorable outcomes, Iwilfin is being evaluated for use in post-immunotherapy or as a maintenance drug with conventional treatment for high-grade neuroblastoma.

However, DFMO has various side effects that make it difficult for patients to adhere to the regimen and for healthcare practitioners to monitor these patients continuously. As a result, more research is needed to reduce the negative effects of DFMO and boost its efficacy in the face of resistance caused by long-term usage. It may be difficult for certain people to afford or acquire Iwilfin treatment, particularly in places with undeveloped healthcare systems. Equitable access to Iwilfin for neuroblastoma patients worldwide is critical, requiring collaboration among researchers, doctors, and regulatory authorities. Further future recommendations include conducting more clinical studies to assess Iwilfin’s involvement in combination treatment regimens and possible synergies with other medicinal drugs. Furthermore, efforts should be made to optimize treatment procedures and reduce treatment-related toxicities to improve patient outcomes and quality of life.

## 6. Conclusion

In summary, the advancement of Iwilfin marks a significant milestone toward enhancing the therapeutic landscape for high-grade neuroblastoma. This new treatment option offers hope for patients with high-grade neuroblastoma, but further research is essential to fully understand and establish its potential benefits and risks.

## Author contributions

**Data curation:** Ayesha Shakeel, Aniqa Baloch, Versha Kumari, Syeda Kainat Zehra Kazmi, Kanza Aftab, Shiza Abid, Amna Syed, Juvairia Yousuf, Muhammad Hasanain, Muhammad Umair Anjum.

**Funding acquisition:** Mohammed Mahmmoud Fadelallah Eljack.

**Investigation:** Ayesha Shakeel, Aniqa Baloch, Versha Kumari, Syeda Kainat Zehra Kazmi, Kanza Aftab, Juvairia Yousuf, Muhammad Hasanain, Muhammad Umair Anjum.

**Methodology:** Ayesha Shakeel, Aniqa Baloch, Versha Kumari, Syeda Kainat Zehra Kazmi, Kanza Aftab, Shiza Abid, Amna Syed.

**Project administration:** Shiza Abid, Juvairia Yousuf, Muhammad Hasanain, Muhammad Umair Anjum.

**Resources:** Juvairia Yousuf.

**Supervision:** Juvairia Yousuf, Muhammad Hasanain, Muhammad Umair Anjum.

**Validation:** Ayesha Shakeel, Aniqa Baloch, Versha Kumari, Syeda Kainat Zehra Kazmi, Kanza Aftab, Shiza Abid, Amna Syed, Juvairia Yousuf, Muhammad Hasanain, Muhammad Umair Anjum.

**Writing – original draft:** Ayesha Shakeel, Aniqa Baloch, Versha Kumari, Syeda Kainat Zehra Kazmi, Kanza Aftab, Shiza Abid, Amna Syed.

**Writing – review & editing:** Juvairia Yousuf, Muhammad Hasanain, Muhammad Umair Anjum.
